# GP-led adapted comprehensive geriatric assessment for frail older people: a multi-methods evaluation of the ‘Living Well Assessment’ quality improvement project in Scotland

**DOI:** 10.3399/BJGPO.2022.0184

**Published:** 2023-02-08

**Authors:** Eddie Donaghy, Franca Still, Helen Frost, Julia Lutte, Susan D Shenkin, Helen E Jones, Stewart W Mercer

**Affiliations:** 1 Usher Institute, University of Edinburgh, Edinburgh, UK; 2 Medical School, University of Edinburgh, Edinburgh, UK; 3 Penicuik Medical Practice, Midlothian, Penicuik, UK; 4 Department of Medicine of the Elderly, Western General Hospital, NHS Lothian, Edinburgh, UK; 5 Advanced Care Research Centre, Usher Institute, University of Edinburgh, Edinburgh, UK

**Keywords:** comprehensive geriatric assessment, frailty, aged, general practice, primary healthcare

## Abstract

**Background:**

Evidence to support comprehensive geriatric assessment (CGA) in primary care for frail older people is limited.

**Aim:**

To evaluate a GP-led adapted CGA quality improvement project.

**Design & setting:**

Multi-methods evaluation in a large practice in Midlothian in Scotland.

**Method:**

The intervention was conducted by 10 GPs in a practice of approximately 11 000 patients, initially in the patient’s home, and then remotely (by telephone or video consultation) during the COVID-19 pandemic. Evaluation included a patient questionnaire, and qualitative interviews with GPs delivering the Living Well Assessment (LWA), analysed by thematic analysis.

**Results:**

A total of 165/220 (75%) patients responded to the survey, of which 86% reported a ‘very good experience’ of the LWA. The method of delivery did not significantly influence this although most (58%) stated a preference for face-to-face consultation. For the 31% who preferred remote LWA, most (23%) preferred telephone to video consultation (8%). Problems in remote consultations related to technical issues (video), poor vision (video), or deafness (telephone or video). GPs felt that home-based LWAs had real benefits but switching to remote during the pandemic had proven feasible. Concerns included potential increase in GP workload owing to the LWA and whether it was an efficient use of GPs’ time.

**Conclusion:**

GP-led adapted CGA was feasible in a large practice, even during the pandemic, and highly valued by frail patients. Questions regarding efficient use of GPs’ time, effectiveness in terms of important patient outcomes and impact, and cost-effectiveness, requires further investigation in a larger study.

## How this fits in

As populations age and develop multimorbidity, the prevalence of frailty is likely to increase substantially. CGA is well established as an evidence-based intervention in frailty in secondary care, and although there is some evidence of effectiveness of community-based CGA, this has mainly been in geriatrician-led community services, and there is very limited evidence on GP-led CGA.

In this multi-methods evaluation of a GP-led, adapted CGA quality improvement project, patients reported a ‘very good experience’ of the LWA, irrespective of whether it was face-to-face at home or conducted remotely. This study has found that GP-led adapted CGA is feasible and highly valued by frail patients, but further research is needed on the role of the multidisciplinary primary care team, and the effectiveness and cost-effectiveness of such an approach.

## Introduction

Frailty has been recognised as a major public health issue worldwide.^1^ Frailty is ‘*a state of vulnerability due to age-related decline in multiple physiological systems*’,^2^ which leaves the body unable to respond effectively to illness or injury.^3,4^ Frailty is associated with an increased risk of hospitalisation, reduced functional ability, falls, and death.^5^ The UK NHS spends approximately £6 billion a year on frailty care.^6^ Furthermore, if frailty goes unaddressed, patients can undergo excessive, invasive treatments when facing unanticipated health crises, which may not be in keeping with their preferences or adding value to their care.^7^

Frailty is associated with ageing and multimorbidity, both of which are rapidly increasing.^8^ However, frailty is not inevitable,^9^ and a proactive approach to identifying and better managing patients at risk of the adverse effects of frailty could result in valuable improvements to patients.^2^ The evidence base surrounding frailty management is expanding because of its pressing relevance.^10^ Research on the identification, assessment, and management of frailty in the setting of secondary care is well established^2,11^ with the CGA being widely accepted as the ‘gold standard’ for the management of frail patients in hospital.^2^ The CGA is a holistic process that appraises the physical, psychosocial, functional, and environmental needs of frail patients.^12^

Primary care^13^ provides an ideal setting for routinely identifying high-risk patients,^14^ but there is no established model of frailty assessment and management,^15^ and limited evidence of effectiveness or cost-effectiveness of community-based CGA,^12,15–17^ even though an adapted CGA for use within general practice is recommended by the British Geriatrics Society.^18^ Barriers to implementation need further investigation.^19^ Research on community-based CGA shows wide variation in who conducts the assessment, including geriatric specialists,^19,20^ nurses, physiotherapists, and social workers.^19,21,22^ There are very few studies that have evaluated CGA delivered by GPs.^19^ Furthermore, following the start of the COVID-19 pandemic, which saw the rapid implementation of telemedicine methods for most primary care patients in the UK, evidence regarding the experience of remote CGAs is even more limited. A study carried out before the COVID-19 pandemic found remote CGAs to be reliable, compared with face-to-face CGA, in making clinical triage decisions.^23^

This present study evaluated a quality improvement project that was conducted in Penicuik Medical Practice, which is a large (>11 000 patients) GP practice in the Midlothian county of Scotland. The number of peopled aged ≥75 years in Midlothian is expected to double by 2035 and the cost of care provision to this group increased by over £1 million during 2016 alone.^24^ Thus, frailty management and ongoing quality improvement for frailty care within the health board led to Midlothian Health and Social Care Partnership (HSCP) supporting the practice in introducing a new model of care in August 2019, based on an adapted CGA, called the Living Well Assessment (LWA).

Understanding the provider and patient experience of care is crucial to service evaluation and ultimately quality improvement.^25^ Therefore, this study explored the experience of older people who took part in the LWA by means of a bespoke survey; the views of the GPs are also explored in qualitative interviews and focus groups.

## Method

The overall aim of this multi-methods study was to:

1. evaluate the impact of the LWA quality improvement project in primary care from the GPs’ and patients’ perspectives;
2. determine whether there was a preference in the methods of delivery of LWA (face to face and remote [telephone or video]).

### Intervention and setting

The LWA is a GP-led intervention of adapted CGA for patients living at home with moderate or severe frailty, designed as a quality improvement project to deliver proactive goal-oriented and patient-centred care to better support patients with frailty in the community. The practice is the larger two situated in the town of Penicuik, in the Midlothian region of Scotland, which has a population of approximately 16 500 people. Penicuik is within easy commuting distance to Edinburgh. Penicuik developed into a town mainly because of local paper mills in the late 18^th^ century, and then through nearby coal mining, which ended in the late 19^th^ century. Penicuik is mixed in terms of demographics. It has a wide range of people, housing and activities, and a mix of professional and non-professional residents. People with higher and lower educational attainment of the age and deprivation profile of the practice list is comparable with the average for Scotland, although the practice size is larger (>11 000, versus the Scottish average of 6325).^26^ At the time of the project, there were six GP partners in the practice and four salaried GPs, all of whom took part in delivering the LWAs. The practice also had two GP trainees and a two-session a week academic GP, who did not take part in delivering the LWAs. The practice also employs several practice nurses and healthcare assistants, who did not take part in the LWAs.

The LWA developed with financial support from the Midlothian HSCP. The participating GPs received training and guidance from JL, who led the project, having herself received training from a practice in the North of Scotland which had implemented a similar approach. Patients who were identified as moderately or severely frail by the electronic frailty index (a score of >0.24)^27,28^ and living at home (that is, not in a care home) received the LWA by a GP (10 GPs shared the assessments between them) in the practice. The assessment was guided by a checklist for consistency, which was based on the toolkit published by the British Geriatric Society^18^ (see supplementary file). The participating GPs received training before the start of the LWAs. Within this 1-hour extended face-to face assessment, patients were able to define their own health needs and consider their medications and treatment. They were also encouraged to discuss an anticipatory care plan (part of the checklist, see supplementary file), a record of patient wishes in the event of deterioration in health, which was then recorded in the electronic Key Information Summary (KIS), which is available to services outside the practice including secondary care and the ambulance service.^29^ Subsequently, further arrangements could be made, such as referrals or medication changes, with support from a multidisciplinary team. Patients with additional needs were discussed at a multidisciplinary team meeting held in the practice once a month.

Initially, doctors visited the patient at home to perform the adapted CGA known as the LWA. However, to be able to continue the assessments throughout the COVID-19 pandemic, remote assessments were used instead. Patients were offered either telephone or video-call assessments depending on available technology. Where required or requested, relatives and/or carers were present.

### Patient survey

Between August 2019 and March 2021, all patients (*n* = 220) who had received a LWA were sent a questionnaire to assess their overall experience of it. An initial questionnaire was piloted in a frailty intervention in a neighbouring practice that targeted the same patient group, and changes were made following feedback from academic clinicians and local GPs (see supplementary file for final version sent to patients). Questionnaires were sent by an administrator within the practice with a pre-addressed, pre-stamped return envelope (to the practice) in March 2021 to all participants who had participated in the LWA between August 2019 and March 2021. The instructions for completion of the questionnaire encouraged people to use help, if needed. Reminder questionnaires to non-responders were sent after 4 weeks. Postcodes were used to calculate patient decile ranking according to the Scottish Index of Multiple Deprivation (SIMD).^30^ Only the administrator had access to the patient’s details. When the questionnaires were returned, the envelopes were discarded by the administrator and the anonymous questionnaire placed in a sealed box. FS then collected these completed questionnaires at regular intervals and entered the data into SPSS while in the practice. FS conducted this work as a medical student at the University of Edinburgh, supervised by SWM, SS, and HJ.

SPSS (version 24) was used for data analysis. The χ^2^ test of association was used to analyse associations between two or more categorical variables. Owing to the non-parametric distribution of data, the Mann–Whitney *U*-test was used to look for significant differences in patient scores according to differing groups of a variable and the Kruskal–Wallis test was employed when variables were split into more than two groups.

Thematic analysis^31^ was used to explore the free text from the questionnaire open-ended responses. Comments were transcribed verbatim on a separate document and coded. The codes were then studied for similarities to identify any recurring themes from the feedback by FS, and discussed with SWM and ED, who are both experienced qualitative researchers. This was done iteratively, over several meetings, before final agreement on the themes were reached.

### Interviews and focus groups with GPs

The 10 GPs delivering the LWA gave one-to-one interviews and focus group interviews. Nine one-to-one interviews were completed: four face to face, and (because of COVID-19 restrictions) five by telephone using a semi-structured interview topic guide. They were conducted by an experienced qualitative health services researcher (ED) during March 2020, at which time the LWAs were being carried out by the GPs in the patients' homes. The interview guide is shown in the supplementary file.

As a result of lockdown, two focus groups were then organised with the 10 GPs in September 2020 via Microsoft Teams. Focus groups were used rather than individual interviews because of the increased workload and disruption to the practice caused by COVID-19. Conducting focus groups was a pragmatic decision as it would have been difficult to schedule individual interviews. This time, the LWAs were also carried out by telephone or video. Focus group 1 (*n* = 7) lasted 45 minutes and focus group 2 (*n* = 3) lasted 40 minutes. All interviews and focus groups were recorded on an encrypted audio-recorder and transcribed verbatim. The interview guide is shown in the supplementary file.

A thematic analysis approach to the data was used.^31^ ED and SWM independently developed initial codes based on individually analysing several transcripts and agreed on the coding frame through discussion. The transcripts were then coded by researcher ED. The codes were then studied for similarities and data were analysed thematically to extract key themes.^31^ Agreement on the key themes was reached in an iterative manner, through several regular meetings between ED and SWM. NVivo software (version 12 Pro) was used for the qualitative analysis. Consolidated criteria for reporting qualitative research (COREQ) guidelines were followed for conducting and reporting the qualitative component of this study.^32^ Results were shared and feedback was requested from the GPs who led the LWA project, and responses allowed for refinement of emerging findings. In terms of the positionality of the researchers and how they interacted with the GPs during the research, ED who conducted the interviews and led the analysis, is a non-clinical senior qualitative university lecturer and had no interaction with the GPs other than arranging and conducting the interviews. SWM, however, is an academic GP who has worked in the practice 1 day a week for many years, and thus knows the GPs well. However, he was not involved in delivering the LWA, and ED and SWM were mindful and reflective about this during the analysis.

## Results

### Patient survey

A total of 165 valid responses were received from 220 (75%) of the LWA patients. Almost two-thirds (63%) of patients completed the questionnaire independently. Patients who received help from a relative were more likely to be from the ≥85 year age group (*P* = 0.001).

Table 1 outlines the demographic information collected from responders. The median age group was 85–94 years (46%). The distribution of Scottish Index of Multiple Deprivation (SIMD) decile scores for patients ranged between 3 and 10 (1 being most deprived and 10 least deprived). Table 2 shows the distribution for ‘timing of assessment’ was similar for all four categories. Almost half of patients (47%) received a telephone consultation; the remainder were divided between video call (27%) and home visits (21%). The majority of the home visits (71%) took place 12 months before the start of the COVID-19 pandemic in March 2020. Approximately half of (48%) the phone calls occurred within the last 4 months (from the time of the survey being sent out). The majority (71%) of video calls were between 4–8 months and 8–12 months prior to the survey being sent out (March 2021).

**Table 1.** Patient characteristics of responders who underwent a frailty assessment

Patient characteristics	*n* (%)
Age group, years^a^	
<60	1 (0.6)
60–64	1 (0.6)
65–74	28 (17.0)
75–84	47 (28.4)
85–94	77 (46.0)
≥95	5 (3.0)
Sex^b^	
Male	58 (35.1)
Female	106 (64.2)
SIMD decile^c^(*1* = *most deprived, 10 = least deprived*)	
3	43 (26.1)
4	23 (13.9)
5	14 (8.5)
6	5 (3.0)
7	5 (3.0)
8	31 (18.8)
9	20 (12.1)
10	15 (9.1)

^a^Missing data = 6 (3.6%). ^b^Missing data = 1 (0.6%). ^c^Missing data = 9 (5.5%). SIMD = Scottish Index of Multiple Deprivation

**Table 2.** Details of how and when the assessment was carried out for responders (*n* = 165)

Information on the Living Well Assessment	*n* (%)
Timing of assessment^a,b^	
>12 months ago	41 (24.8)
8–12 months ago	34 (20.6)
4–8 months ago	36 (23.1)
<4 months ago	45 (21.8)
Method of assessment^c^	
Home visit	34 (20.6)
Telephone call	77 (46.6)
Video call	44 (26.6)

^a^Missing data = 9 (5.5%). ^b^From time of survey being sent out. ^c^Missing data = 10 (6.1%).

**Figure 1.**
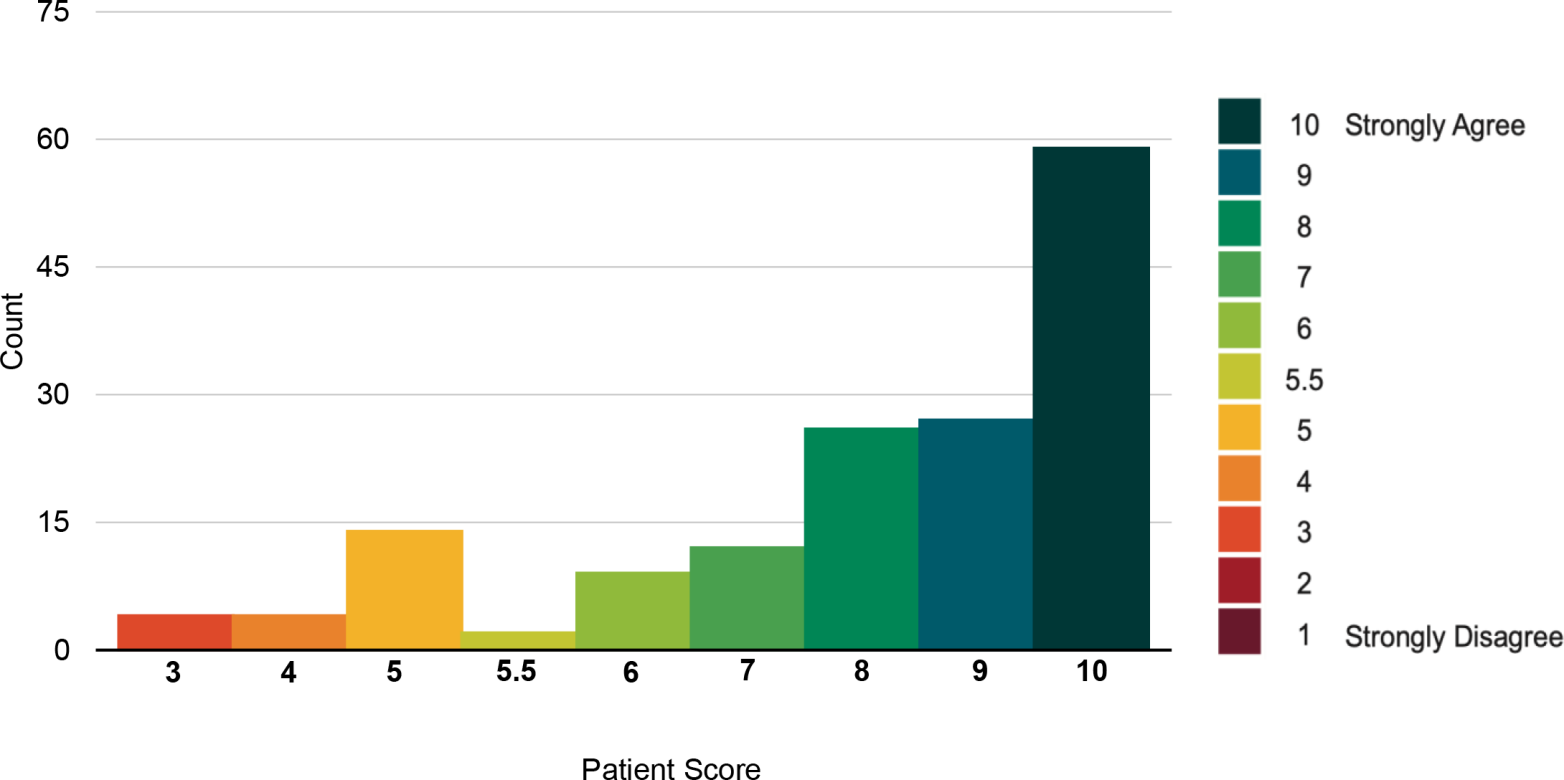
Histogram showing distribution of scores that patients provided regarding their agreement with the statement *'Overall, I had a very good experience of the Living Well Assessment'*. A Mann–Whitney *U*-test concluded that scores for agreement with this statement did not differ with age group (dichotomised), *U* = 2756, *P* = 0.63. Nor did the scores differ by sex, *U* = 2527, *P* = 0.34. A Kruskal–Wallis test found no association between patient score and method of assessment, χ2 (2) = 0.80, *P* = 0.67. Patient agreement and time of assessment were not associated either, χ2 (3) = 1.89, *P* = 0.57. Finally, there was no relationship between SIMD decile and score *U* = 2734, *P* = 0.89.

The response to the statements *'Overall, I had a very good experience of the Living Well Assessment*' and *'I was happy with the type of consultation used for my Living Well Assessment’* showed very high agreement (See Figures 1 2), with a median score of 9 (out of a maximum of 10). Overall, 86% reported a very good experience of the LWA (scoring >5). Only 14% of patients disagreed with the first statement (scoring ≤5).

**Figure 2.**
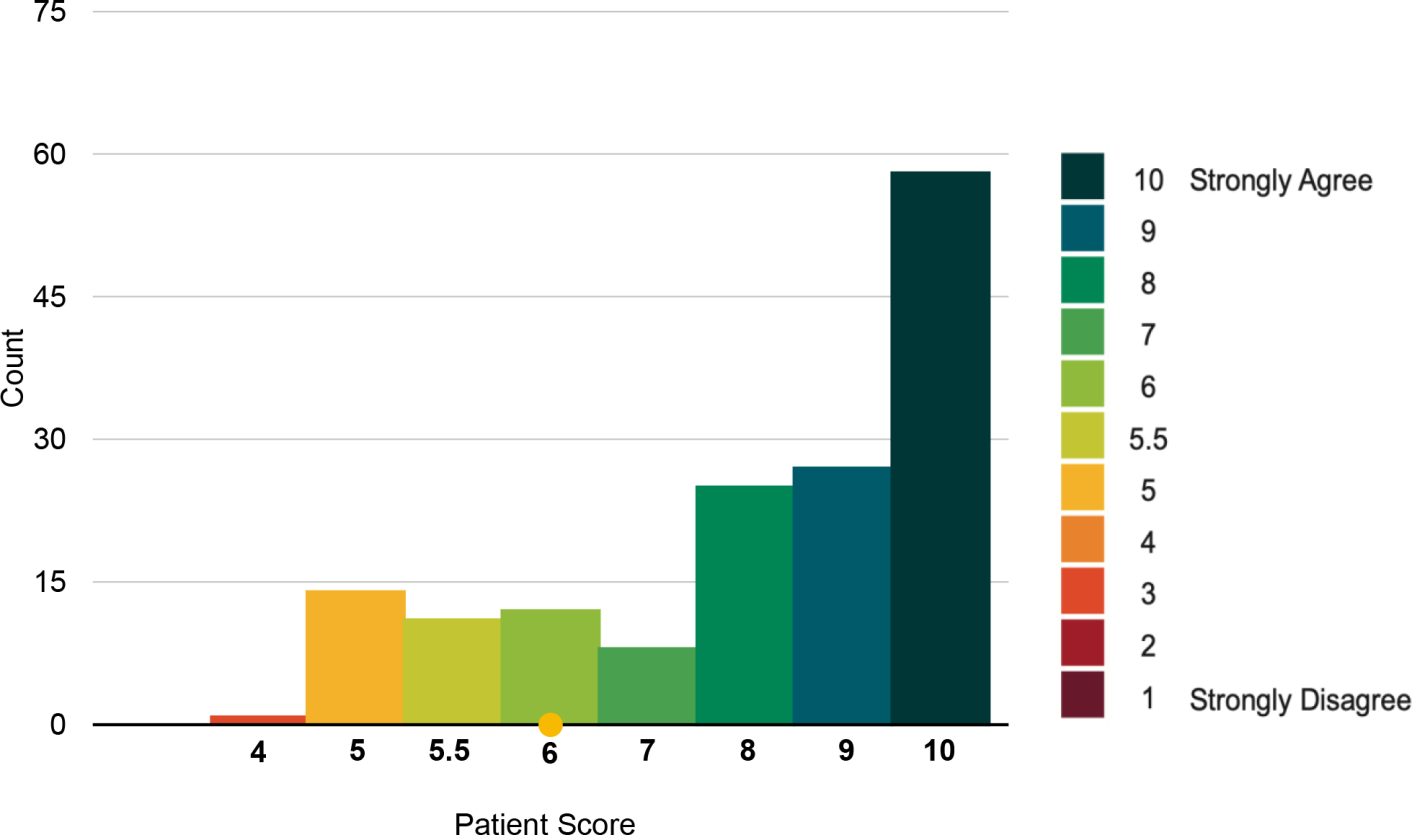
Distribution of scores patients provided regarding their agreement with statement, '*Overall, I was happy with the type of consultation used for my Living Well Assessment*'. Proportionally, a greater number of video-call patients (24.4%) were unhappy with their consultation type, in comparison with telephone patients (13.7%) and home-visit patients (12.1%). The difference in scores according to method was non-significant, χ2 (2, *n* = 147) =4.5, *P* = 0.106.

**Figure 3.**
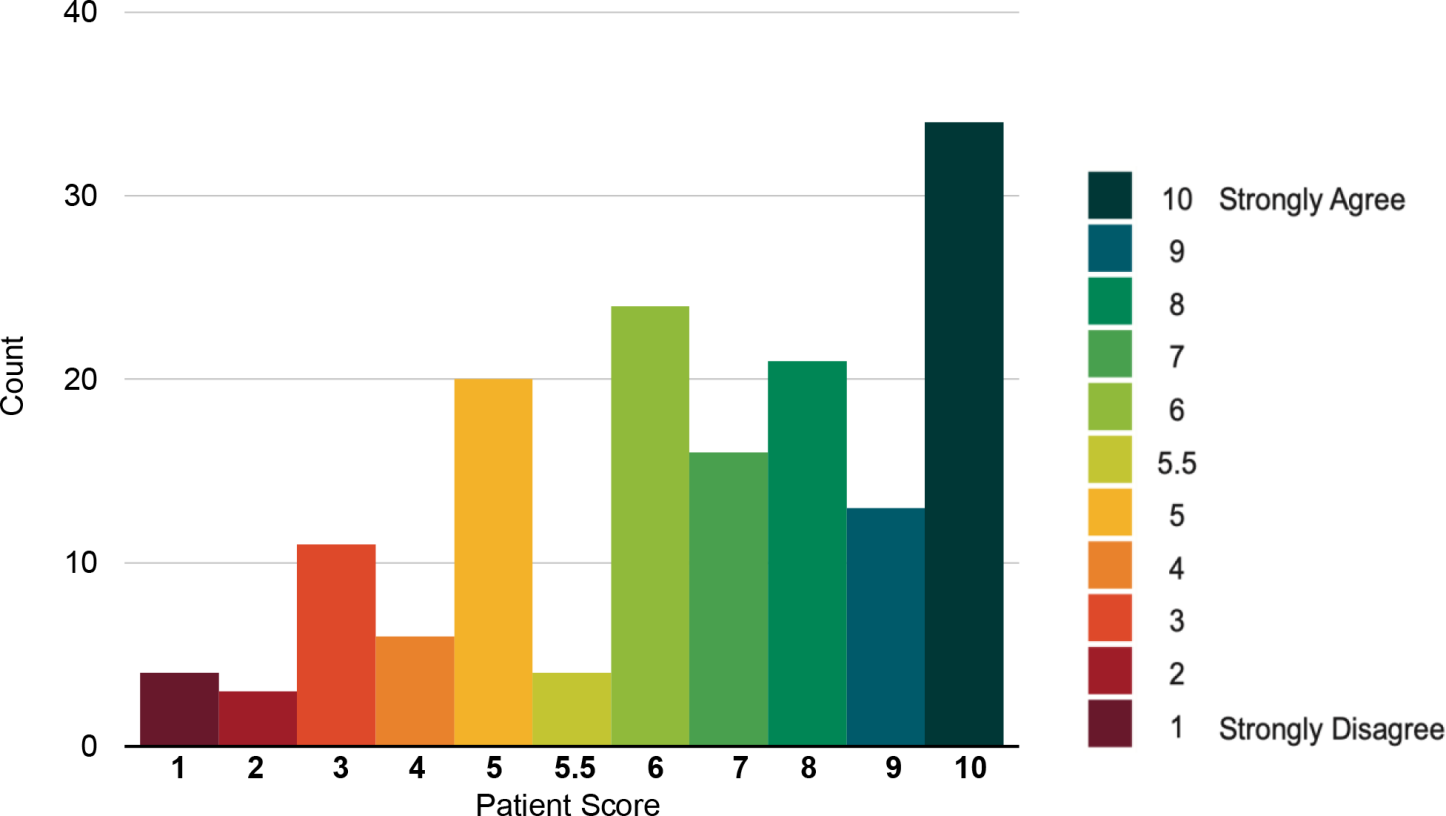
Distribution of scores patients provided regarding their agreement with statement,*''The Living Well Assessment has improved my health care going forward'*

For the statement *'The Living Well Assessment has improved my health care going forward',* the median score was 7 (See Figure 3).

For patients who had a telephone appointment for their LWA, most (75%) did not experience any technology-related issues. Of those (25%) who did, this could be attributed to 16 (84%) experiencing difficulties hearing, two (11%) had concerns about privacy, and one individual (5%) experienced a poor line connection. Notably there were more problems encountered with video-call assessments; in total, 24 (54%) patients experienced difficulties (some had multiple issues) using video with this form of communication. This included the following: 12 patients (50%) requiring help to set up the device used; nine (38%) having difficulty hearing; nine (38%) with limited technological experience; five (21%) with technical issues; and five (21%) having difficulty seeing. Many patients required a family member to facilitate the video call. In terms of future preference , the majority (59%) of patients reported that they would opt for a face-to-face assessment, the most popular being a home visit (31%).

**Figure 4.**
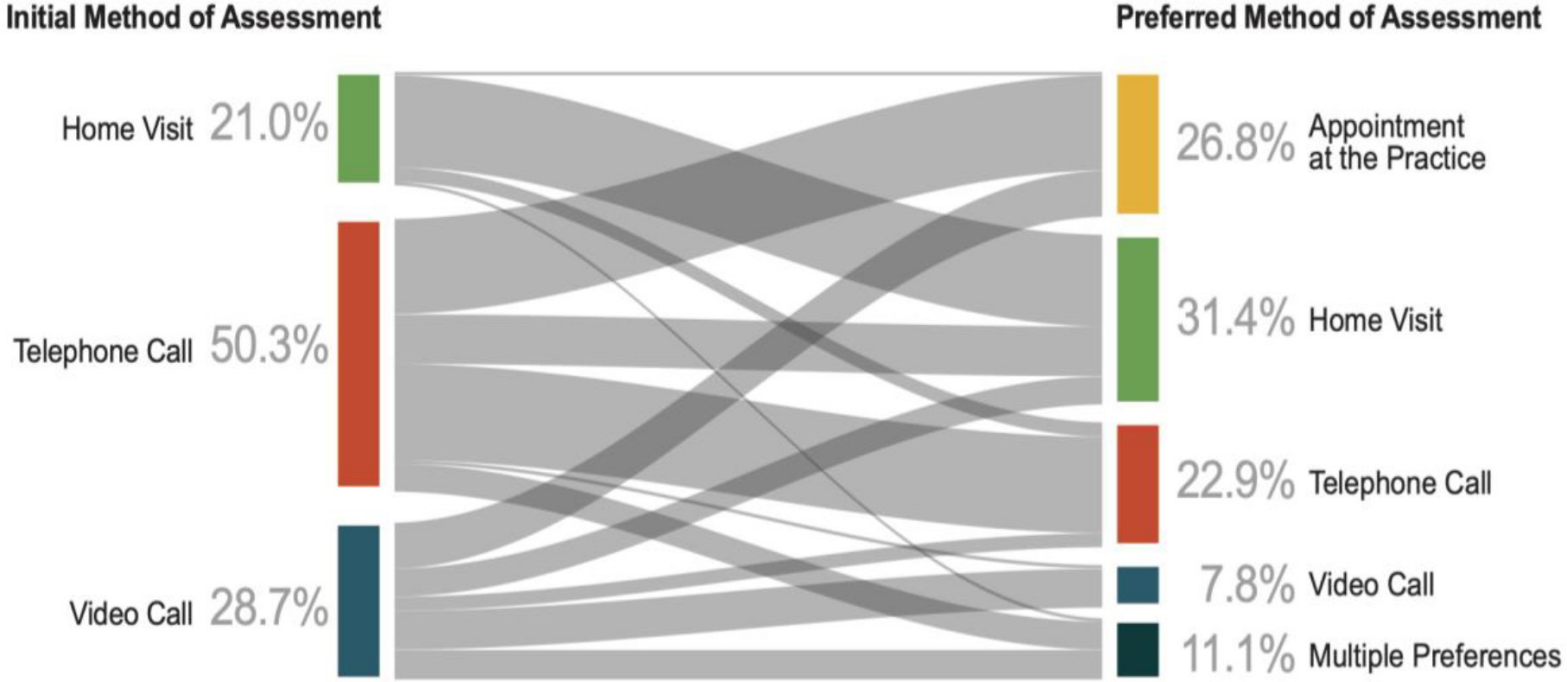
Sankey diagram showing the relationship between initial method of assessment and patient preference for future assessment method

The Sankey diagram (Figure 4) shows the relationship between the initial method of assessment and future preference for method of consultation. In terms of future appointments for LWA, 58% preferred face-to-face, and 31% preferred remote LWA; most (23%) preferred telephone and (8%) video. A significant association was found between initial and preferred assessment type. Patients who had a home visit were more likely to opt for a face-to-face method in the future (*P* = 0.003). Telephone call was the most preferred remote method, chosen by 23% overall, and 35% of patients who had one initially. In the case of 18 (11%) responders, there was an equal preference for two methods. These patients were excluded from the analysis owing to their absolute preference being unknown. The results also showed patient preference for face-to-face methods, with all but one combination opting for one or more form of in-person assessment.

### Data from open-ended questions

Supplementary Table S1 contains the themes and all data identified from the open-ended survey questions that offered insight into aspects of the assessment patients liked, and potential barriers to successful implementation. Themes included are discussed below.

#### Positive response to the LWA

The provision of the assessment made participants feel that their overall wellbeing and general health was being prioritised by the GPs:

*'I appreciated the call from my doctor, and it was good to know that they were checking up on me and asking me important questions about my health.*' (R38)

#### More detailed and lengthier assessments welcomed

The longer appointment time allowed for the discussion of multiple topics and improved the responder experience. The longer assessment also covered more topics in detail than a normal consultation in the GP surgery, and provided an opportunity for the staff to thoroughly discuss and understand the needs of the patient:

*'The assessment was good, in enabling the beginning of a proactive relationship between patient and doctor and resulting in the doctor actually having a personal knowledge — previously lacking.' (R51*)

#### Inadequate follow-up

Some participants expressed disappointment over the absence of a follow-up with a doctor, to provide a conclusion to the assessment:

*'No follow-up action. Think it would have been a good thing to be part of if it had worked how it should have.'* (R51)

#### Face-to-face consulting preferred

Overall, participants expressed the view that a face-to-face consultation is the preferred option:

*'You can't beat a face-to-face examination with a doctor.'* (R45)

#### Disability issues necessitated family involvement

For many responders the presence of a family member improved their experience of the assessment, with many relying on their relative to hear the doctor or use the remote device over which the assessment was taking place. Trouble hearing owing to the device used or personal difficulty with hearing was a common problem:

*'This was good apart from my hearing difficulty.'* (R32)

### GP interviews and focus groups

From the thematic data analysis the following five key themes were identified: (i) initial thoughts and key outcomes of the LWA; (ii) assessment of project’s progress; (iii) benefits of a longer assessment in the patient’s home; (iv) unintended consequences (negative and positive); and (v) key learning, sustainability and roll-out, and are further illuminated in the Supplementary Table S2.

Longer assessments in the patient’s own home had benefits as they allowed the GP to locate the patient’s frailty and the broader LWA in the context of the patient’s immediate social circumstances, as this GP noted:

*'You get to locate their frailty in their home which gives you a much greater insight into how they’re coping. Assessing access to back and front door, trip hazards, what are the stairs like, bathroom access? Medicines storage. They are at home and more relaxed which also makes for a better consultation.*' (PGP7)

Most GPs believed the lengthier home assessments resulted in opportunities for improved advanced care planning completion rates and reduction in polypharmacy:

*'More time with the patient meant I had a chance at a thorough medicines review which could potentially reduce polypharmacy.'* (PGP3)

However, GPs believed that ‘harder’ outcome measures, such as reduced hospitalisations, would be difficult to achieve. GPs who were newer to the practice especially welcomed the opportunity to get to know their patients better through the long home assessments and valued the relationship building that these longer home assessments generated, as this relatively new GP explained:

*'The opportunity to develop a stronger relationship was always a positive outcome for me. To discuss what mattered to them in terms of their health and wellbeing and what they wanted to support them in the future was always very attractive.'* (PGP9)

While lengthier home assessments by GPs had their benefits, GPs raised questions about whether this was an efficient use of GP time and was cost-effective:

*'I did think, wow, that’s a lot of time with one patient, how cost-effective is this?'* (PGP7)

For some GPs, the longer assessment form was too complex. Many GPs believed large parts of the CGA could be completed by non-GPs, making the assessment process less time-consuming and therefore more efficient and more cost-effective. However, GPs believed key clinical decisions based on the lengthier assessment should be made by GPs.

An additional key finding was (i) more time to conduct an in-depth frailty assessment resulted in patients being upgraded from moderate to severely frail, as this GP highlighted:

*'You’ve gone out there to prevent them being severely frail and you’ve instead identified them as severely frail. You uncover new things because you have more time. We’re not coding people as well as we could be. All the people I’ve seen have been coded up and now are severely frail.'* (PGP2)

Key findings also included (ii) GP perceptions of increased workload, and (iii) GP concerns over increased patient expectations:

*'I think one of my initial concerns was about a culture shift. In terms of patient expectation around more referrals to physio and OT* [occupational therapy]*. And patients expecting more house calls as a result of this work.'* (PGP5)

### Key learning and outcomes from video and telephone assessments

While a face-to-face consultation with a GP was seen as the gold standard, GPs felt that LWA by video (when it worked without technical problems) was a feasible and acceptable way of assessing frailty and key ‘Living Well’ issues.

The GPs felt that a LWA by video (without technical problems) could improve anticipatory care planning completion rates and reduce polypharmacy. However, it was also recognised that for some frail older patients, an LWA by video either wasn’t appropriate or could only occur with a family member present:

*'There’s no doubt that there are some barriers to frail people using video. Physical, hearing difficulties, and cataracts. But with the help of relatives, I think people are quite happy to crack on with it.'* (PGP18)

GPs believed that face-to-face contact in the home was superior to video consultation as they were able to better assess the patient’s home circumstances and conduct a physical examination.

*'It’s better to speak to a patient face to face. It feels better for patients. From a GP viewpoint, it’s clinically better to have the patient in front of you*.' (PGP10)

*'Having done 7–8 of these LWAs, the biggest benefit was meeting the patients and getting more detailed knowledge about them both clinically and socially.'* (PGP8)

A few GPs felt that given COVID-19 lockdown restrictions, assessment by video was safer than face-to-face consultations and was likely to continue during lockdown:

*'With COVID, reducing footfall into practice is safer. The other thing in COVID times, video consultations reduced the need for unnecessary PPE to have a conversation.'* (PGP10)

In terms of hierarchy, face-to-face LWAs were accepted as the gold standard. Assessments by video were feasible and acceptable to GPs, especially during lockdown. Telephone consultations were the least favoured of the three assessment mediums:

*'It’s something as simple as smiling, you can see them smiling, they can see you smiling. There’s more of a shot at bedside manner. It’s very difficult to have bedside manner on a telephone. It’s difficult to have bedside manner on a face-to-face consultation with full PPE.'* (PGP17)

Face to face was also the most appropriate for initially raising and discussing sensitive topics such as ‘do not attempt cardiopulmonary resuscitation’ (DNACPR).

## Discussion

### Summary

GP-led LWA was a positive experience for most frail patients and, although the method of delivery did not significantly influence this response, most would prefer a face-to-face consultation, with telephone being the second most popular choice. GPs felt that home-based LWAs had real benefits but switching to remote during the pandemic had proven feasible. Concerns included potential increase in GP workload owing to the LWA and whether it was an efficient use of GPs’ time.

### Strengths and limitations

Strengths of the study included the multi-methods approach and the longitudinal nature of the qualitative interviews with the GPs, which captured views before and after the move to remote consulting prompted by the pandemic, and also the high response rate to the patient survey. Weaknesses included the limited sample size of the qualitative study with the GPs (although all 10 GPs who were delivering the intervention were interviewed, which was the majority of GPs working in the practice). Thus, the authors are confident the views captured were representative of the practice, but, of course, this cannot be extrapolated beyond this in terms of saturation of themes or representativeness of views of GPs in Scotland more widely. Strengths of this study included the high response rate to the survey (75%), and the perspective from patients and GPs. Finally, not having patient and public involvement (PPI) in the design of the study and interpretation of findings was a weakness.

A second weakness was that the patient survey was sent at a single-time point (March 2021) and thus patients had varying periods of time between having the LWA and completing the survey. Recall may have been poorer in those who had the LWA earlier, that is, those who had face-to-face assessments.

### Comparison with existing literature

Rietkerk *et al* explored CGAs in a community setting (but not delivered by the GP or primary care team) in The Netherlands^21^ and found that although frail patients valued the CGA, the effect on healthcare outcomes was limited.^21^ A feasibility study conducted in the US^32^ found high patient and provider satisfaction from geriatrician-led remote CGA and, as in the present study, telephone was more accessible to older people than videos.^33^ However, a UK study found that while face-to-face consultations were seen as the ‘gold standard’, video consultations had some benefits over telephone in terms of visual cues and improving rapport.^34^ A Cochrane review found low-certainty evidence that CGA in a community setting increases the likelihood of frail older people being able to remain at home and reduces the risk of unplanned hospital admission,^12^ but all the trials were geriatrican- rather than GP-led. Another systematc review in community settings reported improvement in function, quality of life, and mental health outcomes following CGA for older frail people.^19^ Most CGAs were conducted by geriatrician or geriatric nurses.^12,19,20^ However, a recent Swedish trial of CGA in primary care carried out by nurses and physicians reported a reduced need for hospital day care for older people at high risk.^17^ There are no direct comparisons of GP-led CGA compared with geriatrician-led CGA. However, in a large Dutch trial there was no difference reported in patient satisfaction between different care providers.^35^ Overall, there is a lack of understanding about how CGA works or what the essential components of this complex intervention are, or which health practitioners are best placed to deliver the intervention.^36,37^

### Implications for research and practice

Further randomised controlled trials are clearly required on the effectiveness and cost-effectiveness of GP-led primary care-based CGA, given the lack of robust evidence on this area. It is recommended that GPs and primary care teams who are considering delivering adapted CGA use a template based on best practice to standardise assessment.^18^

In summary, there is limited evidence on community-based CGA in primary care, especially GP-led CGA, and further evaluation is needed to determine effectiveness and factors relating to successful implementation. Home-based adapted CGA by GPs was highly valued by frail patients. Unsurprisingly, patients and GPs prefer face-to-face consultation, although remote methods were feasible, especially by telephone.

## Data

The authors do not have permission to share the dataset relied on in this article.
